# Oral challenge test with nsaids: evaluation of patients attending a specialty clinic in Ribeirão Preto, Brazil

**DOI:** 10.1186/1939-4551-8-S1-A207

**Published:** 2015-04-08

**Authors:** Ullissis Menezes, Janaina M Melo, Daniel Cordeiro, Priscila Palhas, Karine Boufleur, Juliana Poli, Phelipe Souza, Isabela Mina, L Karla Arruda

**Affiliations:** 1 Clinical Hospital of Ribeirão Preto Medical School, Brazil; 2 Ribeirao Preto Medical School, University of Sao Paulo, Brazil; 3 Brazilian Association of Allergy and Immunology, Brazil

## Background

Non-Steroidal Anti-Inflammatory Drugs (NSAIDs) are the most common cause of non-allergic drug-induced systemic hypersensitivity reactions. We have aimed to use the Oral Challenge Test (OCT) to determine alternative and safe drugs to be used by patients with hypersensitivity to NSAIDs, and evaluate its most prevalent reactions.

## Methods

Prospective study including 116 patients with positive history of hypersensitivity to NSAIDs, evaluated at the Allergy Clinic of the Clinical Hospital of Ribeirao Preto Medical School, between October 2010 and July 2014. Patients were evaluated using the European Network for Drug Allergy questionnaire, and underwent single blind, placebo controlled OCT, in a hospital-controlled environment. Patients were required to be clinically stable, and to withdraw antihistamines or corticosteroids seven days prior to the OCT. Patients were given 10%, 20%, 30% and 40% of the therapeutic NSAID dosage, selecting an NSAID different from the one implicated in the allergic reaction. Fifteen minutes after each dosage, patients were evaluated with Peak Flow and blood pressure measurements, heart and respiratory rates and general examination.

## Results

Among the 116 patients enrolled the study, 85(73%) were females. Eighty-five patients(73%) had history of allergic respiratory disease. Clinical manifestations were: Angioedema (58%), Urticaria(42%), Anaphylaxis(24%), and respiratory symptoms such as nasal pruritus, sneezing, rhinorrhea, dyspnea, and cough (22%). The NSAIDs most implicated by history were: Dipirone(70%), Diclofenac(52%), Acetaminophen(29%), Ibuprofen(21%), Cetoprofen(20%) and Nimesulide(19%). Reactions to two or more drugs were reported by 86 patients(74%). One-hundred and fifty-three OCT were performed, with 28(18%) positive results. The most implicated drugs were Celecoxib (7 tests), Benzydamine(5), Nimesulide(3), Ibuprofen(3), ASA(2), Acetaminophen(2), Etoricoxib(2), Dipirone(1), Diclofenac(1), Meloxicam(1), and Viminol(1). Reactions included Angioedema, Urticaria and respiratory symptoms. One patient presented with anaphylaxis during OCT using Celecoxib.

## Conclusions

It is well established that COX-2 inhibitors are safe and low-reactive drugs, recommended as an alternative to patients with hypersensitivity to NSAIDs, however in this study we have observed reactions involving Celecoxib. OCT showed to be an effective method to find an alternative and safe choice to the patients with NSAIDs hypersensitivity.

